# Development and validation of a prognostic model to predict the prognosis of patients who underwent chemotherapy and resection of pancreatic adenocarcinoma: a large international population-based cohort study 

**DOI:** 10.1186/s12916-019-1304-y

**Published:** 2019-03-25

**Authors:** Lei Huang, Yesilda Balavarca, Lydia van der Geest, Valery Lemmens, Liesbet Van Eycken, Harlinde De Schutter, Tom B. Johannesen, Vesna Zadnik, Maja Primic-Žakelj, Margit Mägi, Robert Grützmann, Marc G. Besselink, Petra Schrotz-King, Hermann Brenner, Lina Jansen

**Affiliations:** 1 Division of Clinical Epidemiology and Aging Research, German Cancer Research Center (DKFZ), Im Neuenheimer Feld 581, 69120 Heidelberg, Germany; 2 Medical Faculty Heidelberg, Heidelberg University, Heidelberg, Germany; 3 Division of Preventive Oncology, German Cancer Research Center (DKFZ) and National Center for Tumor Diseases (NCT), Heidelberg, Germany; 4 Netherlands Cancer Registry (NCR), Netherlands Comprehensive Cancer Organization (IKNL), Utrecht, The Netherlands; 5 Belgian Cancer Registry (BCR), Brussels, Belgium; 6 Registry Department, The Cancer Registry of Norway (CRN), Oslo, Norway; 7 Epidemiology and Cancer Registry, Institute of Oncology Ljubljana, Ljubljana, Slovenia; 8 Estonian Cancer Registry, National Institute for Health Development, Tallinn, Estonia; 9 Department of Surgery, Friedrich-Alexander-University Erlangen-Nürnberg, Erlangen, Germany; 10 Dutch Pancreatic Cancer Group (DPCG), Department of Surgery, Cancer Center Amsterdam, Amsterdam University Medical Centers (UMC), Academic Medical Center (AMC), University of Amsterdam, Amsterdam, The Netherlands; 11 German Cancer Consortium (DKTK), German Cancer Research Center (DKFZ), Heidelberg, Germany

**Keywords:** Pancreatic cancer, Resection, Chemotherapy, Survival, Prognostic factors, Benchmark population-based nomogram, International real-world cohort study

## Abstract

**Background:**

Pancreatic cancer (PaC) remains extremely lethal worldwide even after resection. PaC resection rates are low, making prognostic studies in resected PaC difficult. This large international population-based study aimed at exploring factors associated with survival in patients with resected TNM stage I–II PaC receiving chemotherapy and at developing and internationally validating a survival-predicting model.

**Methods:**

Data of stage I–II PaC patients resected and receiving chemotherapy in 2003–2014 were obtained from the national cancer registries of Belgium, the Netherlands, Slovenia, and Norway, and the US Surveillance, Epidemiology, and End Results (SEER)-18 Program. Multivariable Cox proportional hazards models were constructed to investigate the associations of patient and tumor characteristics with overall survival, and *analysis was performed in each country respectively without pooling*. Prognostic factors remaining after backward selection in SEER-18 were used to build a nomogram, which was subjected to bootstrap internal validation and external validation using the European datasets.

**Results:**

A total of 11,837 resected PaC patients were analyzed, with median survival time of 18–23 months and 3-year survival rates of 21–31%. In the main analysis, patient age, tumor T stage, N stage, and differentiation were associated with survival across most countries, with country-specific association patterns and strengths. However, tumor location was mostly not significantly associated with survival. Resection margin, hospital type, tumor size, positive and harvested lymph node number, lymph node ratio, and comorbidity number were associated with survival in certain countries where the information was available. A median survival time- and 1-, 2-, 3-, and 5-year survival probability-predictive nomogram incorporating the backward-selected variables in the main analysis was established. It fits each European national cohort similarly well. Calibration curves showed very good agreement between nomogram-prediction and actual observation. The concordance index of the nomogram (0.60) was significantly higher than that of the T and N stage-based model (0.56) for predicting survival.

**Conclusions:**

In these large international population-based cohorts, patients with resected PaC receiving chemotherapy have distinct characteristics independently associated with survival, with country-specific patterns and strengths. A robust benchmark population-based survival-predicting model is established and internationally validated. Like previous models predicting survival in resected PaC, our nomogram performs modestly.

**Electronic supplementary material:**

The online version of this article (10.1186/s12916-019-1304-y) contains supplementary material, which is available to authorized users.

## Background

Worldwide, pancreatic cancer (PaC) is the seventh leading cause of cancer-related mortality [1]. Resection remains the cornerstone of curative treatment for medically fit patients with resectable locoregional PaC [2]. However, only a small proportion of patients with PaC undergo resection. In the United States (US) and Europe during 2012–2014, resection rates ranged from 13% (Estonia) to 21% (Slovenia) for all-stage PaC and from 35% (Norway) to 69% (Denmark) for stage I–II PaC [3]. The 3-year overall survival remains poor even for patients with stages I–II PaC who underwent resection (< 60 years, 23–39%; 60–69 years, 16–31%; and ≥ 70 years, 17–30%) [4]. Chemotherapy has been routinely recommended for resected PaC [5–7]; however, it remains challenging to get many patients to adjuvant therapy after pancreatectomy [8].

Various prognostic factors for PaC including clinical/pathological (e.g., tumor stage, size, differentiation, lymph node status, resection margin, and peri-neural and blood vessel invasion [9–15]), genetic (e.g., *KRAS*, *TP53*, *SMAD4*, and some DNA damage repair genes (e.g., *BRCA1/2* and *MLH1*) [16–18]), and immunological variables (e.g., CD3, CD8, CD68, PD-L1, and HHLA2 [19–21]) have been reported. Further large international comparative studies on survival-associated factors at the *population* level could help to identify differences across countries.

Patients with resected PaC who undergo chemotherapy are a selected group of all PaC patients and have distinct characteristics [8]. Even within this patient group, survival is heterogeneous. A prognostic model for this specific patient population is important and desirable and could facilitate clinical counseling by informing both patients and doctors of predicted individualized patient survival, guide plans on follow-up and surveillance, aid to survival stratification in international studies, and offer the baseline survival estimates for further molecular or genetic investigations. Furthermore, for resected patients considering subsequent chemotherapy, the predicted results could potentially encourage a proportion of patients with specific characteristics to further receive the standard postsurgical care. Stage is the major prognostic factor for PaC. Notably, survival of patients with disease of the same TNM stage might vary greatly [14]. Other prognostic factors such as patient age and tumor differentiation could improve individualized survival-prediction. A model incorporating all these factors can be intuitively illustrated using a nomogram [22]. Apart from two institutional nomograms predicting postsurgical survival in overall patients [23, 24], *population-based* survival-predicting models specifically for resected PaC patients receiving chemotherapy with international validations and robustness have not been found.

To our knowledge, we herein report the first large international population-based investigation into factors associated with survival in patients with resected TNM stage I–II PaC receiving chemotherapy. We further construct a population-based survival-predicting model with international validations.

## Methods

### Patients

Population-based data of resected PaC patients were obtained from the national cancer registries of Belgium, the Netherlands, Slovenia, and Norway, and the US Surveillance, Epidemiology, and End Results (SEER)-18 [25] database. Data quality was previously described [3]. Institution-based data were not included due to the highly selected patients. An extensive attempt was made to contact population-based cancer registries, and the contacted registries together with reasons for exclusion are shown in Additional file 1: Table S1. The participating European national registries, located in Western, Southern, and Northern Europe were those able to provide quality data according to a standardized uniformed data-request form, which ensured the robustness of the results. All variables were uniformly (re)coded across registries. While there were other national population-based registries, they were not always able to provide eligible treatment, TNM staging, or survival data. All patient-level data were anonymous. This real-world observational study was approved by the Ethics Committee of Medical Faculty Heidelberg.

Patients with diagnosis based on death certificate only (DCO)/autopsy or with unknown/obscure follow-up time or vital status were excluded (Additional file 1: Table S2). Only patients with microscopically confirmed diagnoses of primary invasive TNM stage I–II adenocarcinomas of the exocrine pancreas who underwent surgical resection in 2003 until 2014 were selected. The time period was selected based on data availability and the fact that the fifth and prior editions of TNM staging were incompatible with the sixth/seventh versions used during 2003–2017 [7]. Since chemotherapy is standard for patients with resected PaC [5–7], we only included those receiving chemotherapy. Individuals with benign/premalignant tumors, non-PaC neoplasms involving the pancreas, neuroendocrine tumors/carcinoids, cystic/mucinous/serous tumors, acinar cell tumors, stromal tumors, sarcomas, germ-cell neoplasms, lymphomas, or peri-ampullar tumors were also excluded (Additional file 1: Table S3). To minimize the effect of the potential heterogeneity in surgery quality and perioperative care, we excluded cases surviving < 3 months. Patients with stage III or IV PaC were also excluded since resection is not routinely recommended for these patients [5–7].

Information on demographic (sex and age), clinical (year of diagnosis/surgery and treatment), and pathologic characteristics (topology, morphology, and TNM stage) was retrieved from all participating countries. Data on resection margin (the Netherlands and Slovenia), hospital type (Belgium and the Netherlands), Eastern Cooperative Oncology Group (ECOG) performance status score (Belgium), comorbidities (Eindhoven, the Netherlands), resection type (the US and the Netherlands), tumor size (the US), and positive and harvested lymph node numbers (the US and the Netherlands) were only available in certain registries.

Resection was defined as surgical removal of primary tumor, regardless of being curative or palliative and extents of excision and lymphadenectomy. Tumor topography and morphology were based on the International Classification of Diseases for Oncology (third edition). Stage was defined following the TNM staging system (sixth/seventh edition) and was a combination of pathologic and clinical stages with priority given to pathologic staging. Lymph node ratio was calculated by dividing the number of positive lymph nodes by the number of harvested lymph nodes. Vital status was based on valid national mortality registrations and official population registers.

### Statistical analyses

*Data in each country were analyzed separately without pooling*, considering the potential heterogeneity across countries and to avoid the impact of any single large cohort. Descriptive results were reported as the smallest to the largest proportions for categorical variables or medians/means for continuous variables across countries. The cancer incidence rates by sex in each country were retrieved from the Cancer Incidence in Five Continents Volume XI (CI5 XI) by the International Agency for Research on Cancer (IARC), World Health Organization (WHO) (http://ci5.iarc.fr/CI5-XI/Default.aspx), which reports the incidence of cancers diagnosed from 2008 to 2012, standardized to the World (WHO 2000–2025) Standard Population.

The Kaplan-Meier method was applied to calculate survival time and rates. Since patients surviving < 3 months were excluded in this study, the 6- and 9-month survival was calculated as the short-term outcome. The 1-, 2-, 3-, and 5-year survival was computed as the long-term outcome. To assess the independent impact of potential prognostic factors on survival, Cox proportional hazards regression was used. Variables including year of diagnosis, age, sex, tumor location, T and N stages, and differentiation were included as covariates in the main multivariable models. For complete-case analysis, patients with missing data were excluded in multivariable analyses. In the US, results for the white patients were computed for comparison with the total patients, for whom main analyses were performed. In registries with available information, resection margin, hospital type, tumor size, positive and harvested lymph node numbers, lymph node ratio, T and N stages according to the eighth edition following Kamarajah et al. [26], ECOG score, resection type, and comorbidities were incorporated one by one into the main models to examine the survival association for each of them. The proportional hazards assumption was verified for all variables by plotting the logarithm of the negative logarithm of the survival function against the logarithm of survival time [27].

Data were centrally analyzed in the German Cancer Research Center. Results were considered statistically significant at two-sided *P* < 0.05. Analyses were conducted using the SAS software (version 9.4, SAS Institute Inc.).

### Nomogram construction and validation

The SEER-18 dataset, the largest of the included datasets, was used as the training set for nomogram construction (models based on the other cohorts did not reveal markedly better performance). Age, sex, tumor location, T and N stages, and differentiation were entered as potentially relevant prognostic factors into the initial full multivariable Cox proportional hazards regression model, and the final model was selected through a backward step-down process using the likelihood ratio test with the Akaike information criterion as a stopping rule [28]. To permit nonlinear associations, continuous variables were modeled using restricted cubic splines where appropriate [28]. Points assigned to each variable included in the nomogram to predict the median survival time and 1-, 2-, 3-, and 5-year survival probability were proportional to the effect size of that variable in the final multivariable model. To facilitate clinical use, a corresponding online prognostic tool was created with Evidencio (https://www.evidencio.com/).

The nomogram was subjected to 1000 bootstrap resamples for internal validation of the training US cohort and was externally validated using the European datasets to assess the international generalizability of the model. The model performance and discrimination ability for predicting survival was numerically evaluated by computing Harrell’s concordance index (C-index) [28]. Comparison of C-indexes of different models followed Hanley et al. [29]. Calibration of the nomogram for 1-, 2-, 3-, and 5-year survival was done by comparing the predicted with the observed survival. Bootstrapping was used for bias correction [28].

In sensitivity analyses for the training US cohort, C-indexes were re-calculated after replacing continuous age with age group, N stage with positive lymph node number or lymph node ratio, and sixth/seventh edition of cancer stages with the eighth version, after adding harvested lymph node number and/or tumor size, after limiting patients to those diagnosed after 2009 or white patients, and after stratifying patients by tumor location. The *survival* and *rms* packages in R 3.4.1 (http://www.r-project.org) were used.

## Results

### Patient characteristics

In total, 168,949 PaC patients were registered in the population-based registries in 2003/2004–2013/2014 with follow-up until 2015–2016. After excluding patients diagnosed based on DCO/autopsy (*n* = 4403), unresected (*n* = 137,605), receiving no/unknown chemotherapy (*n* = 11,465), with microscopically unconfirmed tumors or with tumors of ineligible pathology (*n* = 1418), with stage 0/III/IV/unknown tumors (*n* = 1856), and with survival< 3 months or unknown (*n* = 365), 11,837 patients were eligible for analysis (Additional file 1: Table S2). The detailed counts and frequencies for discrete variables and medians and interquartile ranges for continuous variables are shown in Table 1. Age-standardized PaC incidence was higher for males than for females. Among the participating countries, incidence for males was lowest in Belgium and the Netherlands (7.4 per 100,000) and highest in Slovenia (9.3 per 100,000); for females, incidence was lowest in Belgium (5.6 per 100,000) and highest in the US, Slovenia, and Norway (6.5 per 100,000).

**Table 1:** Demographic and clinical characteristics of resected pancreatic cancer patients^1^

Variable	Category	The US	Belgium	The Netherlands	Slovenia	Norway
Incidence (per 100,000)^2^	Male	8.6	7.4	7.4	9.3	7.8
	Female	6.5	5.6	6.1	6.5	6.5
Years of diagnosis		2004–2015	2004–2013	2003–2014	2003–2013	2003–2014
*n*		9519	1105	982	118	113
Diagnosis in 2010 or later	Yes	5635 (59)	579 (52)	747 (76)	67 (57)	79 (70)
Sex	Female	4671 (49)	522 (47)	483 (49)	50 (42)	58 (51)
Age (years)	Median (interquartile range)	65 (58–72)	65 (58–71)	64 (57–69)	61 (54–68)	63 (59–70)
	Mean ± standard deviation	65 ± 10	64 ± 10	62 ± 9	61 ± 9	64 ± 8
	< 50	706 (7)	90 (8)	92 (9)	11 (9)	4 (4)
	50–59	2101 (22)	264 (24)	235 (24)	38 (32)	29 (26)
	60–69	3464 (36)	406 (37)	417 (42)	46 (39)	50 (44)
	≥ 70	3248 (34)	345 (31)	238 (24)	23 (19)	30 (27)
Tumor location^3^	Pancreas head	7314 (83)	658 (82)	820 (90)	97 (92)	91 (88)
	Pancreas body	622 (7)	58 (7)	31 (3)	5 (5)	4 (4)
	Pancreas tail	845 (10)	86 (11)	63 (7)	3 (3)	8 (8)
	Other	738 (8)	303 (27)	68 (7)	13 (11)	10 (9)
T stage^4^	T1	494 (5)	56 (5)	72 (7)	0 (0)	8 (8)
	T2	1192 (13)	185 (17)	182 (19)	8 (7)	28 (26)
	T3	7815 (82)	860 (78)	727 (74)	108 (93)	70 (66)
N stage^5^	N1	6339 (67)	805 (73)	703 (72)	97 (84)	60 (55)
Differentiation^6^	Well	858 (10)	149 (15)	91 (11)	12 (11)	3 (3)
	Intermediate	4540 (52)	511 (52)	423 (51)	44 (40)	64 (63)
	Poor/undifferentiated	3266 (38)	326 (33)	319 (38)	55 (50)	35 (34)

^1^Categorical data are shown as count (percentage [%]). For brevity, results for the counterparts in dichotomous variables are omitted. Records are complete otherwise specified below

^2^The cancer incidence rates by sex in each country were retrieved from the Cancer Incidence in Five Continents Volume XI (CI5 XI) by the International Agency for Research on Cancer (IARC), World Health Organization (WHO) which reports the incidence of cancers diagnosed from 2008 to 2012, standardized to the World (WHO 2000–2025) Standard Population

^3^The percentages of pancreas head, body, tail, and overlapping cancers are the proportions compared to the total tumor cases of the four locations; “other” includes overlapping lesion, pancreas duct, and not otherwise specified location, and its proportion is relative to the whole cases

^4^Missing T stage: the US: 18 (< 1%); Belgium: 4 (< 1%); the Netherlands: 1 (< 1%); Slovenia: 2 (2%); Norway: 7 (6%)

^5^Missing N stage: the US: 0 (0%); Belgium: 7 (1%); the Netherlands: 0 (0%); Slovenia: 2 (2%); Norway: 3 (3%)

^6^Missing differentiation: the US: 855 (9%); Belgium: 119 (11%); the Netherlands: 149 (15%); Slovenia: 7 (6%); Norway: 11 (10%)

Of the analyzed patients, 52–76% were diagnosed in 2010 or later. The proportion of women ranged from 42% (Slovenia) to 51% (Norway) across countries. While the proportion of women was almost identical to that of men in the US, the Netherlands, and Norway, there was a smaller proportion of women in Slovenia (42%). The median age ranged from 61 (Slovenia) to 65 years (the US and Belgium) across countries. Most patients were ≥ 60 years (58% (Slovenia) to 71% (Norway)). The proportion of patients aged ≥ 70 years was greatest in the US (34%) and smallest in Slovenia (19%). Only 4% (Norway) to 9% (the Netherlands and Slovenia) of patients were < 50 years old. Tumors were most commonly located at pancreas head (82% (Belgium) to 92% (Slovenia)). Only 3% (the Netherlands) to 7% of cancers (the US and Belgium) were located at pancreas body and 3% (Slovenia) to 11% (Belgium) at pancreas tail. Only a minority of patients had T1 (0% (Slovenia) to 8% (Norway)) or T2 cancers (7% (Slovenia) to 26% (Norway)). N1 tumors comprised 55% (Norway) to 84% of all cancers (Slovenia). Most patients had either moderately differentiated (40% (Slovenia) to 63% (Norway)) or poorly differentiated/undifferentiated tumors (33% (Belgium) to 50% (Slovenia)). Only 3% (Norway) to 15% of cancers (Belgium) were well-differentiated.

### Survival outcomes

**Fig. 1:**
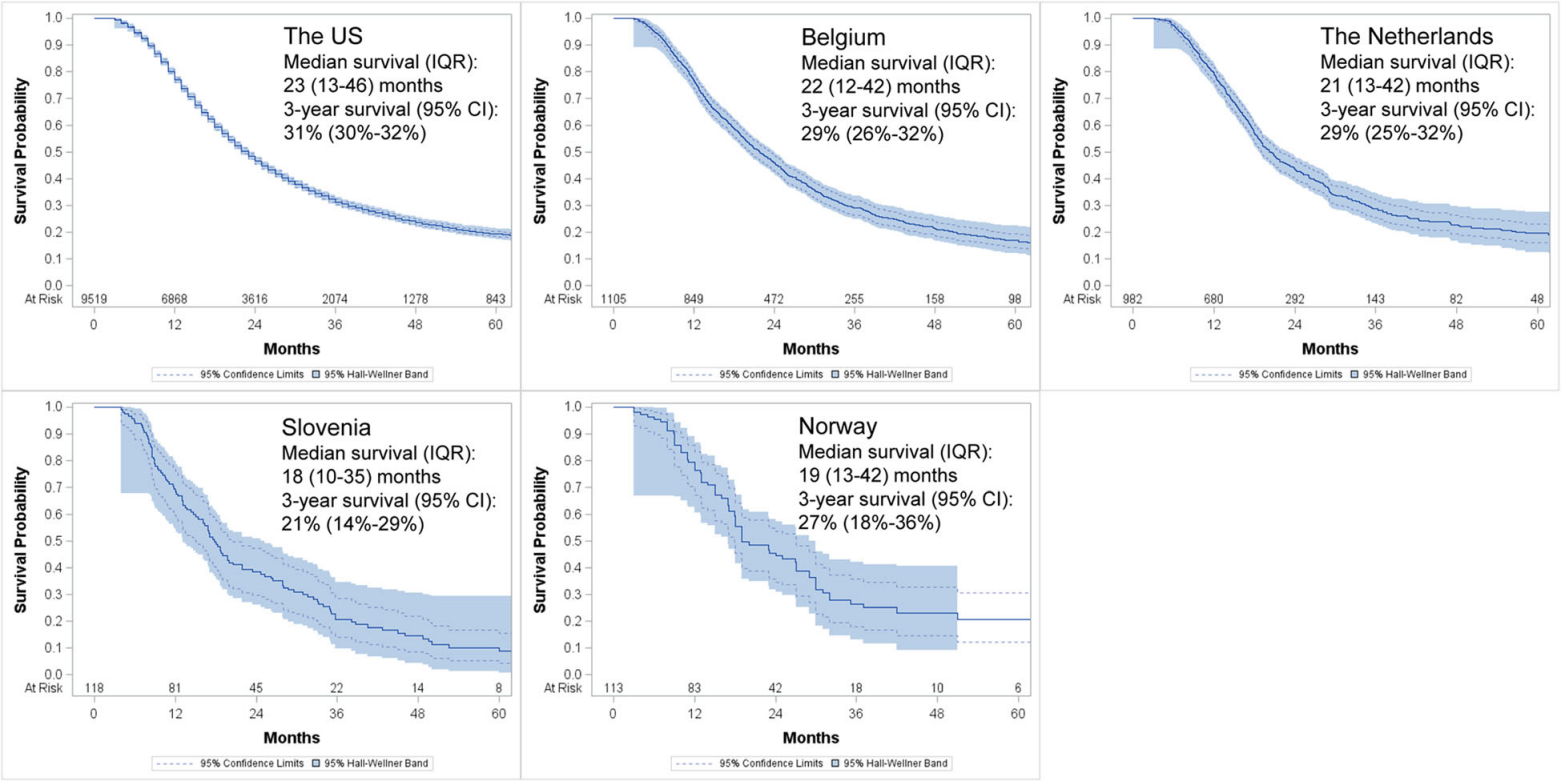
Kaplan-Meier overall survival curves for patients with resected stage I–II pancreatic cancer receiving chemotherapy in each country. The 95% confidence limits curves and the 95% Hall-Wellner bands are additionally shown. Median survival time (interquartile range) in months and 3-year survival rates (95% confidence interval) are calculated and provided. IQR, interquartile range; CI, confidence interval

The median survival time ranged from 18 (Slovenia) to 23 months (the US) across countries (Fig. 1). The short- and long-term survival outcomes are shown in Table 2. The 6-month survival rate ranged from 94% (the US) to 97% (the Netherlands), and the 9-month survival rate varied from 79% (Slovenia) to 90% (Norway). Regarding longer term outcomes, the 1-year survival rate ranged from 69% (Slovenia) to 79% (the Netherlands), and the 3-year survival rate ranged from 21% (Slovenia) to 31% (the US). The 5-year survival rate was lowest in Slovenia (10%), which was about half of that in the US (19%), the Netherlands (20%), or Norway (21%).

**Table 2:** Short- and long-term survival for resected pancreatic cancer patients receiving chemotherapy estimated by the Kaplan-Meier method

Survival	The US	Belgium	The Netherlands	Slovenia	Norway
	OS (95% CI)^1^	OS (95% CI)	OS (95% CI)	OS (95% CI)	OS (95% CI)
Short-term survival					
6-month	94 (94–95)	95 (94–96)	97 (96–98)	95 (89–98)	96 (91–99)
9-month	87 (86–87)	86 (84–88)	89 (87–91)	79 (70–85)	90 (83–94)
Long-term survival					
1-year	77 (76–78)	77 (74–79)	79 (77–82)	69 (60–77)	77 (67–83)
2-year	47 (46–48)	46 (43–49)	44 (40–47)	39 (30–47)	46 (36–55)
3-year	31 (30–32)	29 (26–32)	29 (25–32)	21 (14–29)	27 (18–36)
5-year	19 (18–20)	17 (14–20)	20 (16–23)	10 (5–17)	21 (12–31)

^1^Results were calculated using the Kaplan-Meier method and are shown as survival proportions (95% confidence intervals) [%]

*OS* overall survival, *CI* confidence interval

### Survival-associated factors

Results from multivariable Cox regression are shown in Table 3, and only significant results are described. Increasing age was associated with worse survival in the US (HR per year = 1.01), Belgium (HR = 1.02), and Norway (HR = 1.04). Survival was significantly worse in men only in the US (HR = 1.10) and in pancreas body compared to head tumors in Norway (HR = 2.67). Compared to T3 cancers, T1 cancers were associated with higher survival in all investigated countries (HR = 0.17–0.70), while T2 cancers were associated with better survival only in the US (HR = 0.86). Negative nodal status was associated with significantly higher survival in the US (HR = 0.65), Belgium (HR = 0.78), and the Netherlands (HR = 0.51). Better differentiation was significantly associated with higher survival in all countries except Slovenia and Norway, and the HRs for well- and intermediately versus poorly/undifferentiated tumors were 0.48–0.68 and 0.61–0.81, respectively. Association patterns and strengths were similar between white and overall US patients.

**Table 3:** Association of demographic and clinical variables with overall survival for resected pancreatic cancer patients estimated by adjusted multivariable Cox proportional hazards regression

Variable	Category	The US	The US (white)	Belgium	The Netherlands	Slovenia	Norway
Used no.		8657	7170	979	833	109	96
		HR (95% CI)^1^	HR (95% CI)	HR (95% CI)	HR (95% CI)	HR (95% CI)	HR (95% CI)
Year of diagnosis	Per year; continuous	*0.97* (0.96–0.98)	*0.97* (0.96–0.98)	0.98 (0.95–1.01)	0.99 (0.95–1.03)	0.96 (0.89–1.03)	*0.80* (0.69–0.93)
Age	Per year; continuous	*1.01* (1.01–1.01)	*1.01* (1.01–1.01)	*1.02* (1.01–1.02)	1.00 (0.99–1.01)	1.01 (0.99–1.04)	*1.04* (1.00–1.08)
Sex	Female	1.00 (reference)	1.00 (reference)	1.00 (reference)	1.00 (reference)	1.00 (reference)	1.00 (reference)
	Male	*1.10* (1.05–1.16)	*1.12* (1.06–1.18)	0.93 (0.80–1.07)	1.08 (0.91–1.29)	1.49 (0.96–2.32)	1.14 (0.67–1.95)
Tumor location	Pancreas head	1.00 (reference)	1.00 (reference)	1.00 (reference)	1.00 (reference)	1.00 (reference)	1.00 (reference)
	Pancreas body	1.03 (0.92–1.15)	1.04 (0.92–1.17)	1.34 (0.99–1.82)	1.19 (0.69–2.04)	1.33 (0.47–3.81)	0.40 (0.05–2.98)
	Pancreas tail	1.02 (0.93–1.12)	1.03 (0.93–1.14)	1.00 (0.76–1.30)	0.85 (0.57–1.26)	0.39 (0.09–1.66)	*2.67* (1.09–6.53)
	Other^2^	1.02 (0.93–1.13)	1.03 (0.93–1.15)	0.95 (0.80–1.12)	0.92 (0.65–1.38)	0.83 (0.37–1.84)	0.89 (0.30–2.65)
T stage	T1	*0.66* (0.57–0.75)	*0.70* (0.61–0.81)	*0.68* (0.47–0.97)	*0.48* (0.33–0.71)	–	*0.17* (0.04–0.72)
	T2	*0.86* (0.79–0.93)	*0.88* (0.81–0.97)	0.89 (0.74–1.08)	1.02 (0.82–1.26)	0.70 (0.29–1.67)	0.89 (0.49–1.61)
	T3	1.00 (reference)	1.00 (reference)	1.00 (reference)	1.00 (reference)	1.00 (reference)	1.00 (reference)
N stage	N0	*0.65* (0.61–0.69)	*0.65* (0.61–0.69)	*0.78* (0.66–0.92)	*0.51 (0.41–0.64)*	0.77 (0.40–1.51)	0.71 (0.39–1.29)
	N1	1.00 (reference)	1.00 (reference)	1.00 (reference)	1.00 (reference)	1.00 (reference)	1.00 (reference)
Differentiation	Well	*0.60* (0.55–0.66)	*0.59* (0.53–0.65)	*0.68* (0.55–0.85)	*0.48* (0.35–0.67)	0.57 (0.27–1.22)	0.31 (0.04–2.58)
	Intermediate	*0.77* (0.73–0.81)	*0.78* (0.73–0.82)	*0.81* (0.69–0.94)	*0.61* (0.50–0.73)	0.82 (0.51–1.32)	0.93 (0.54–1.61)
	Poor/undifferentiated	1.00 (reference)	1.00 (reference)	1.00 (reference)	1.00 (reference)	1.00 (reference)	1.00 (reference)

^1^HRs were calculated by Cox proportional hazard regression with adjustment for year of diagnosis, age, sex, tumor location, T, N, and M stages, histology, and differentiation. In stratified analyses, the stratification factor was omitted from the model. Statistically significant HRs are shown in italics

^2^Other: pancreas duct, overlapping lesion, and not otherwise specified location

*HR* hazard ratio, *CI* confidence interval, −, not available

Associations with further variables were explored in countries with available relevant information (Table 4). In the Netherlands, positive resection margin was associated with worse survival in (HR = 1.36), and resection in academic hospital was associated with better survival (HR = 0.79). In the US, larger tumor size was associated with inferior survival, and replacing T stage according to the sixth/seventh edition with the eighth edition revealed similar association patterns and strengths. In the US and the Netherlands, while increasing metastatic node number (HR per positive lymph node = 1.05 and 1.07) and lymph node ratio (HR = 2.60 and 3.15) were associated with inferior survival, more harvested nodes suggested better survival (both HR per harvested node = 0.99). Following the eighth version of TNM staging, N1 (HR = 1.42 and 1.68) and N2 stages (HR = 1.84 and 2.43) were associated with worse survival compared to N0 stage in the US and the Netherlands. In Eindhoven, the Netherlands, more comorbidities were associated with inferior survival (e.g., HR_≥ 2 vs. 0 comorbidities_ = 1.86).

**Table 4:** Association of survival with potential prognostic factors available in at least one registry for resected pancreatic cancer estimated by adjusted Cox proportional hazard regression

Variable	The US		Belgium		The Netherlands		Slovenia	
	n	HR (95% CI)	n	HR (95% CI)	n	HR (95% CI)	*n*	HR (95% CI)
Resection margin								
Negative	–	–	–	–	637	1.00 (reference)	51	1.00 (reference)
Positive	–	–	–	–	291	*1.36* (1.12–1.65)	34	1.54 (0.82–2.88)
Hospital type								
Non-academic	–	–	497	1.00 (reference)	510	1.00 (reference)	–	–
Academic	–	–	608	0.89 (0.77–1.03)	472	*0.79* (0.66–0.94)	–	–
Tumor size								
≤ 2 cm	1490	1.00 (reference)	–	–	–	–	–	–
2–3 cm	3146	*1.23* (1.12–1.35)	–	–	–	–	–	–
3–4 cm	2487	*1.38* (1.25–1.52)	–	–	–	–	–	–
4–5 cm	1229	*1.60* (1.44–1.78)	–	–	–	–	–	–
> 5 cm	938	*1.56* (1.39–1.75)	–	–	–	–	–	–
T stage (8th version)								
T1	1490	*0.62* (0.57–0.68)	–	–	–	–	–	–
T2	5633	*0.81* (0.76–0.87)	–	–	–	–	–	–
T3	2167	1.00 (reference)	–	–	–	–	–	–
Positive LN number (continuous)	9426	*1.05* (1.04–1.06)	–	–	974	*1.07* (1.04–1.10)	–	–
N stage (8th version)								
N0 (0 positive LNs)	3180	1.00 (reference)	–	–	280	1.00 (reference)	–	–
N1 (1–3 positive LNs)	3885	*1.42* (1.33–1.51)	–	–	416	*1.68* (1.33–2.13)	–	–
N2 (≥ 4 positive LNs)	2244	*1.84* (1.72–1.98)	–	–	278	*2.43* (1.89–3.12)	–	–
Harvested LN number (continuous)	9484	*0.99* (0.99–0.99)	–	–	959	0.99 (0.98–1.00)	–	–
LN ratio (continuous)	9138	*2.60* (2.26–3.00)	–	–	945	*3.15* (2.05–4.84)	–	–
ECOG score								
0	–	–	140	1.00 (reference)	–	–	–	–
1	–	–	662	0.96 (0.76–1.20)	–	–	–	–
≥ 2	–	–	63	1.04 (0.73–1.47)	–	–	–	–
Resection type								
Pancreatoduodenectomy	7108	1.00 (reference)	–	–	877	1.00 (reference)	–	–
Distal pancreatectomy	1142	1.02 (0.92–1.14)	–	–	88	1.33 (0.61–2.91)	–	–
Total pancreatectomy	1102	1.07 (0.99–1.15)	–	–	10	0.98 (0.36–2.65)	–	–
Comorbidity								
Cardiovascular disease (yes *v* no)	–	–	–	–	30/119	1.33 (0.69–2.57)	–	–
Hypertension (yes *v* no)	–	–	–	–	39/110	1.01 (0.59–1.75)	–	–
Diabetes (yes *v* no)	–	–	–	–	33/116	1.34 (0.76–2.38)	–	–
Pulmonary disease (yes *v* no)	–	–	–	–	14/135	1.96 (0.88–4.36)	–	–
Number of comorbidities								
0	–	–	–	–	52	1.00 (reference)	–	–
1	–	–	–	–	48	1.48 (0.84–2.62)	–	–
≥ 2	–	–	–	–	49	*1.86* (1.00–3.46)	–	–

^1^The main Cox proportional hazard regression models adjusted for year of diagnosis, age, sex, tumor location, T, N, and M stages, histology, and differentiation. HRs were calculated after N stage was replaced by metastatic node number (group) or lymph node ratio, or after the other investigated variables were included one by one into the main models. Statistically significant HRs are shown in italics

HR, hazard ratio; CI, confidence interval; LN, lymph node; ECOG, Eastern Cooperative Oncology Group; −, not available

### Prognostic nomogram

#### Construction

**Fig. 2:**
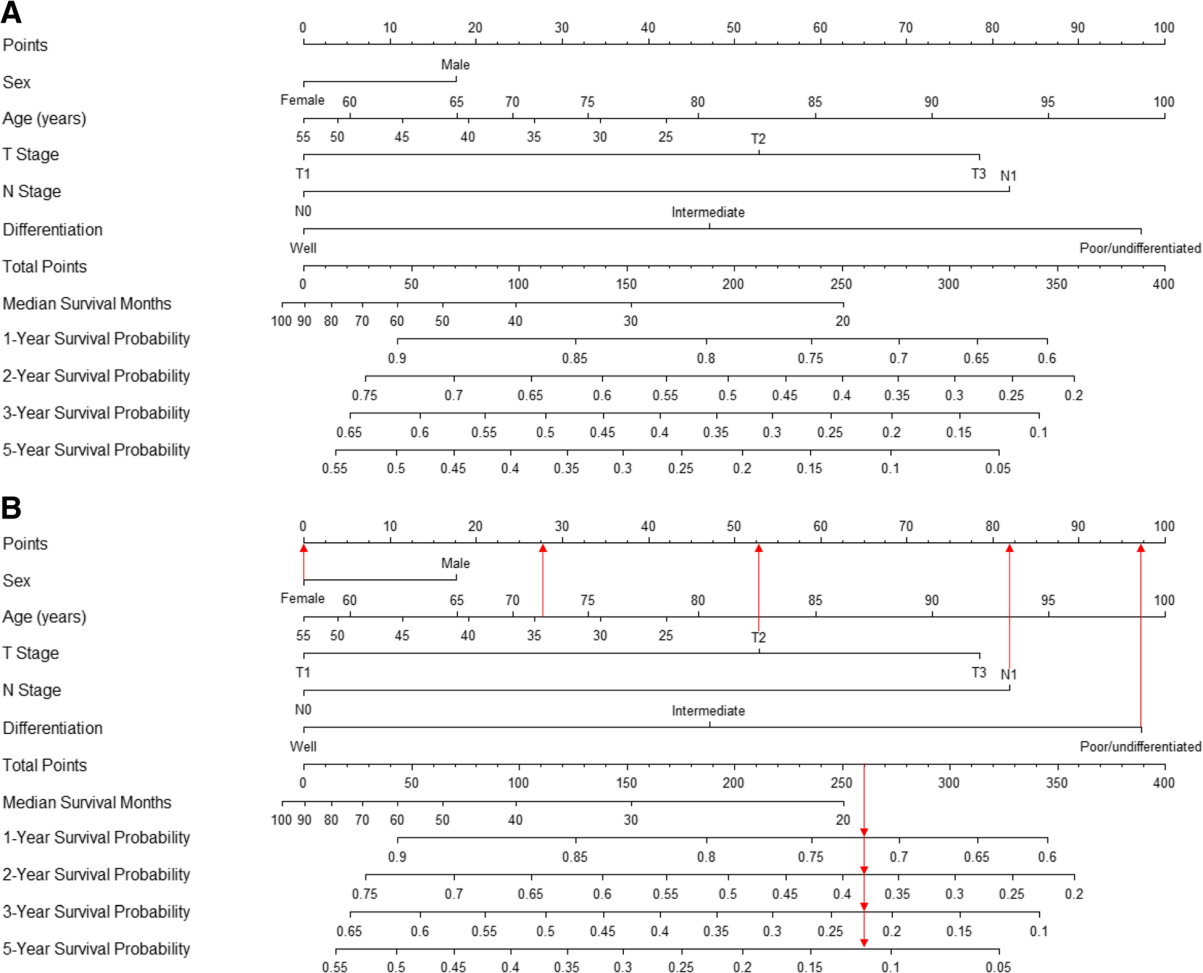
Prognostic nomogram for patients with resected stage I–II pancreatic cancer receiving chemotherapy derived from the US cohort (**a**) and an example on how to use the nomogram (**b**). Each number/category of the prognostic variables is assigned a score on the *Points* scale. After summing up the total score and locating it on the *Total Points* scale, a line drawn straight down to the *Median Survival* or *1-/2-/3-/5-Year Survival* scale shows the median survival time and estimated survival probability at each time point. Age is in years

**Fig. 3:**
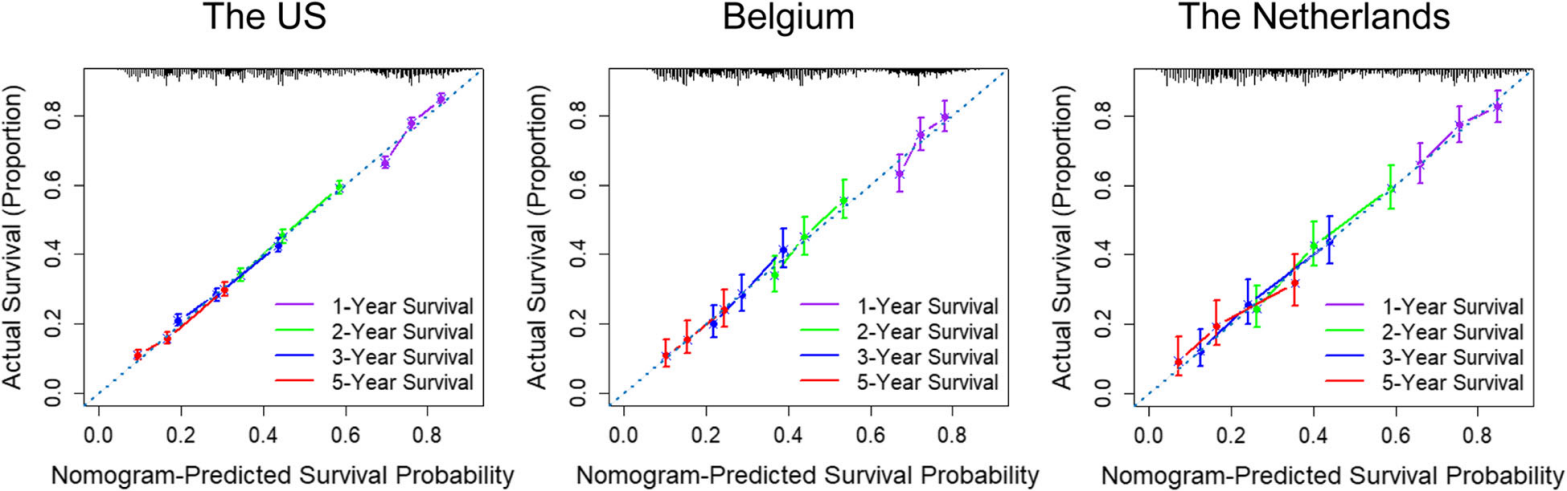
Calibration curves for 1-, 2-, 3-, and 5-year overall survival prediction in the primary training (the US) and validation cohorts (Belgium, the Netherlands, Slovenia, and Norway). Nomogram-predicted survival is plotted on the *x* axis and actual survival on the *y* axis. The vertical bars at the top represent the frequency of the predicted probability of survival. A plot along the 45-degree line indicates a perfect calibration model where the predicted probabilities are identical to the actual proportions

A nomogram incorporating prognostic factors remaining after backward selection in the US (sex, age, T and N stages, and differentiation) was established (Fig. 2a). The nomogram illustrated age and differentiation to have the largest contributions to prognosis. T and N stages showed moderate impacts on survival. Each number/category of these variables is assigned a score on the *Points* scale. After summing up the total score and locating it on the *Total Points* scale, a line drawn straight down to the *Median Survival* or *1-/2-/3-/5-Year Survival Probability* scale shows the estimated survival time or probability at each time point. The model function is provided in the Additional file 1: Supplementary Results. Score assignment for specific categories of the variables and survival for different accumulated scores are shown in Table 5. The layout of an online version of the nomogram is shown in Fig. 3.

**Table 5:** Score assignment for specific categories of the variables included in the nomogram

Prognostic factors		
Variable	Category	Score
Sex	Female	0
	Male	18
Age (years)	25	42
	30	34
	35	27
	40	19
	45	11
	50	4
	55	0
	60	5
	65	18
	70	24
	75	33
	80	46
	85	59
	90	73
	95	86
	100	100
T stage	T1	0
	T2	53
	T3	78
N stage	N0	0
	N1	82
Differentiation	Well	0
	Intermediate	47
	Poor/undifferentiated	97
Median survival		
Total score	Median survival (months)	
251	20	
152	30	
99	40	
64	50	
43	60	
27	70	
13	80	
0	90	
1-year survival		
Total score	1-year survival probability	
345	0.60	
313	0.65	
277	0.70	
236	0.75	
187	0.80	
126	0.85	
44	0.90	
2-year survival		
Total score	2-year survival probability	
358	0.20	
329	0.25	
302	0.30	
276	0.35	
250	0.40	
224	0.45	
197	0.50	
169	0.55	
138	0.60	
106	0.65	
70	0.70	
29	0.75	
3-year survival		
Total score	3-year survival probability	
342	0.10	
305	0.15	
273	0.20	
245	0.25	
218	0.30	
192	0.35	
166	0.40	
139	0.45	
112	0.50	
84	0.55	
54	0.60	
21	0.65	
5-year survival		
Total score	5-year survival probability	
323	0.05	
272	0.10	
235	0.15	
204	0.20	
175	0.25	
148	0.30	
122	0.35	
96	0.40	
70	0.45	
43	0.50	
15	0.55

#### An example of use

An example of how to use the nomogram is shown in Fig. 2b. A 72-year-old woman with poorly differentiated, T2N1M0 PaC who underwent resection and chemotherapy would have 28 points for her age, 0 points for her sex, 53 points for T stage, 82 points for N stage, and 97 points for differentiation, totaling 260 points. The total points correspond to the estimation of median survival time of < 20 months, a 1-year survival probability of 72%, a 2-year survival probability of 38%, a 3-year survival probability of 22%, and a 5-year survival probability of 12%, which are consistent with the results generated by the online tool (Fig. 3).

#### Calibration and validation

**Fig. 4:**
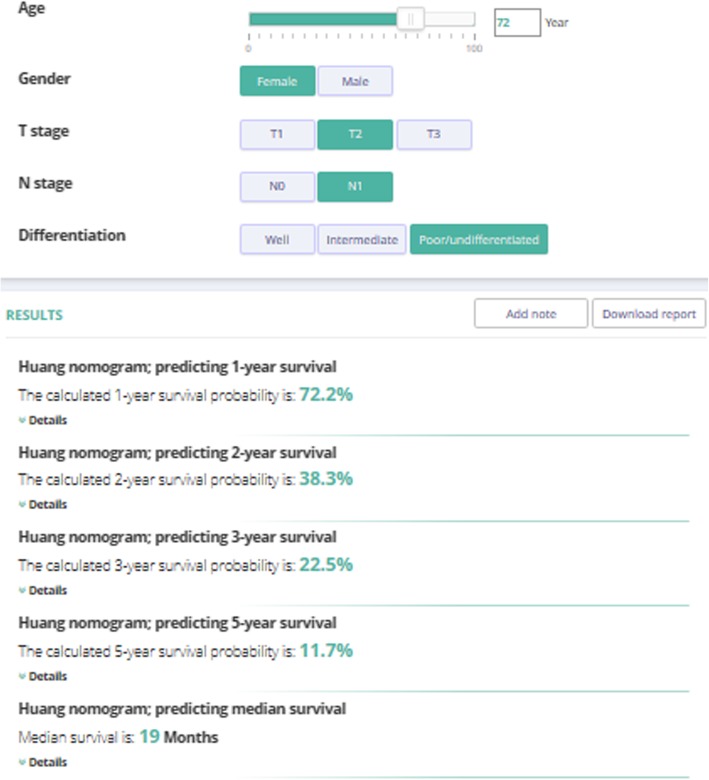
Layout of an online version of the developed nomogram with Evidencio (https://www.evidencio.com/models/show/1258)

The nomogram was applied to the US and the European countries for internal and external validations, respectively. The calibration plots showed very good agreement between nomogram-predicted and actual survival in the US, Belgium, and the Netherlands (Fig. 4; plots were not shown in Slovenia or Norway where the case number was too small to generate meaningful calibration). Generally, the calibration was best for 2- and 3-year survival. In the training US cohort, the C-index for the established nomogram was significantly higher than that for the model based on both T and N stages (0.60, 95% CI = 0.59–0.61 vs. 0.56, 95% CI = 0.56–0.57). In the validation cohorts, C-indexes were also significantly higher for the nomogram than for the T and N stage-based model (Table 6).

**Table 6:** Concordance indexes for resected pancreatic cancer in training and validation cohorts and in sensitivity analyses for the training US cohort

Model modification/subgroup	Concordance index	95% confidence interval
Training cohort		
The US, our nomogram	0.60	0.59–0.61
The US, model based on both T and N stages	0.56	0.56–0.57
Validation cohorts		
Belgium, our nomogram	0.58	0.55–0.60
Belgium, model based on both T and N stages	0.54	0.52–0.56
The Netherlands, our nomogram	0.62	0.59–0.65
The Netherlands, model based on both T and N stages	0.56	0.54–0.59
Slovenia, our nomogram	0.58	0.51–0.65
Slovenia, model based on both T and N stages	0.52	0.47–0.57
Norway, our nomogram	0.63	0.55–0.71
Norway, model based on both T and N stages	0.61	0.54–0.68
Sensitivity analyses for the training US cohort		
Replacement		
Age group in place of continuous age	0.59	0.59–0.60
Metastatic lymph node number in place of N stage	0.60	0.59–0.61
Lymph node ratio in place of N stage	*0.61*	0.61–0.62
The 8th version of T stage in place of the original stage	*0.61*	0.60–0.61
The 8th version of N stage in place of the original stage	0.60	0.59–0.61
The 8th version of T & N stages in place of the original stages	*0.61*	0.60–0.62
Addition		
Harvested lymph node added	0.60	0.60–0.61
Tumor size added	*0.61*	0.60–0.61
Harvested lymph node & tumor size added	*0.61*	0.60–0.62
Subgroup		
Diagnosis after 2009	0.60	0.59–0.61
White ethnicity	0.60	0.59–0.61
Pancreas head	0.60	0.59–0.61
Pancreas body & tail	*0.61*	0.59–0.63

Concordance indexes in sensitivity analyses greater than that for the overall nomogram in the US are highlighted in italics

#### Sensitivity analyses

Sensitivity analyses were performed for the derivative US cohort (Table 6). Using positive lymph node number or lymph node ratio instead of N stage in the nomogram did not obviously change the C-index (by 0.00 and + 0.01, respectively). Replacing the sixth/seventh version of both T and N stages with the eighth version also had minimal impact on the C-index (by + 0.01). After including examined lymph node number, tumor size, or both, the C-index only changed by 0.0, + 0.01, and + 0.01, respectively. Limiting the sample to patients diagnosed after 2009 or white people did not change the C-index. Within subgroups according to tumor location, C-index was slightly higher than the overall one in body/tail cancer (0.61).

## Discussion

In our large population-based study, we identified various factors independently associated with survival after resection of PaC and for the first time established and internationally validated a population-based nomogram for predicting survival in resected PaC patients receiving chemotherapy, which is robust, accurate, reliable, and practical. However, like previous models [23, 24, 30–32], our model had a modest C-statistic.

There are various reports on the prognostic factors for patients who underwent resection for PaC [9–15]. A systematic review showed that with the exception of postsurgical blood transfusion, tumor characteristics (e.g., size, lymph node status, and differentiation) were the only features significantly associated with survival after pancreatic resection [9]. Particularly, PaC size > 2 cm was an independent factor associated with poor post-surgical prognosis [10], and this category has been incorporated in both the sixth/seventh and the eighth TNM staging systems [33, 34]. Notably, neural invasion was also determined to be an independent prognostic factor in PaC [11]. Through multivariable analyses, we demonstrated that older age, more advanced T and N stages, and poorer differentiation were independently associated with lower overall survival in resected PaC across most countries. In registries with available information, resection margin, hospital type, tumor size, metastatic and harvested lymph node numbers, lymph node ratio, and comorbidity number were also associated with prognosis. These findings are mostly consistent with previous literature [9–15, 35, 36] and add insights into the association strengths for resected PaC patients receiving chemotherapy at the population level and into the comparisons between countries. Some patient (e.g., age and comorbidities) and clinical characteristics (e.g., hospital type) were further identified to be prognostically significant. While previous studies have drawn differing conclusions regarding the association between resection type and survival [35, 36], our population-based investigation of chemotherapy-treated resected PaC patients did not show a significant association. Furthermore, we found mostly no significant associations between tumor location and survival.

Notably, overall, the contribution of T or N stage to postoperative survival was mostly not greater than that of differentiation. Categorization of tumor size and number of metastatic lymph nodes following the eighth TNM staging system [33, 34] discriminated survival well, supporting the implementation of the new system. Notably, harvested lymph node number was positively associated with survival. The relevance of harvested lymph node number for survival has remained controversial in PaC [37, 38]. Possible reasons supporting the positive association include that more metastasized lymph nodes may be removed with more extensive sampling, which also results in more precise staging, guiding appropriate post-surgical treatment.

Estimating mortality risk might impact treatment planning and provide information helpful for patient stratification in study design, contributing to better equivalence between study arms [39]. Post-surgical survival for patients with PaC is remarkably heterogeneous, even with the same TNM stage [14, 40, 41]. To our knowledge, the nomogram we developed is the first one derived from a large population-based database with long-term follow-up for predicting overall survival in patients with resected stage I–II PaC receiving chemotherapy, with international validations in multiple European national datasets. There is a previous institutional nomogram [23] developed by Memorial Sloan-Kettering Cancer Center (MSKCC) in 2004 for predicting post-surgical survival in Western PaC patients not accounting for chemotherapy, with three external institutional validation attempts [30–32]. Based on institutional patient cohorts diagnosed many years ago [23, 30–32], the score assignment of several variables might not be optimal currently using the MSKCC nomogram, which might also be limited in generalizability. The MSKCC nomogram did not employ a backward selection process and incorporated some detailed surgical (e.g., portal vein resection and splenectomy) and symptom parameters (back pain and weight loss). Notably, portal vein resection and splenectomy might not be routine procedures during pancreatectomy, and reporting of symptoms might show great interpersonal variations. Our population-based nomogram thus represents a more updated prognostic model compared to the MSKCC nomogram (Additional file 1: Table S5). The wide geographical distribution of patients and large sample size further enhanced the international representativeness and generalizability of our nomogram.

Resection margin, which reflects the radicality of surgery, has not received a universal standard definition in PaC [42, 43], and its relevance for survival remains highly controversial [44, 45]. While we showed a positive association of survival with negative margin in the Netherlands, the strength was not greater than that of the association with T stage, N stage, or differentiation. We did not incorporate this variable in our nomogram for better generalizability. It is encouraged to incorporate margin status into our nomogram when a standard definition comes.

Calibration plots demonstrated very good agreement between nomogram-predicted and actual survival, which assures the repeatability and reliability of our nomogram. Importantly, the model based on the US dataset also fits the multiple European national cohorts, which supports the potential for the generalization and international utilization of our nomogram, *irrespective of the potential health care disparity across countries*. Discrimination of the nomogram, as highlighted by the C-index, was significantly and markedly higher compared to the model based on T and N stages only. In the external validation cohorts, the discriminative potency only slightly changed. Our model performed similarly well across countries, potentially facilitating patient allocation in international studies.

In sensitivity analyses, we examined various alternative models by for instance incorporating positive lymph node number or lymph node ratio as a continuous variable in place of N stage into the nomogram, and the discrimination ability basically remained the same, supporting the robustness of our model.

Notably, the eighth edition of TNM staging system has been implemented since 2018 [33, 34]. Compared to the sixth/seventh version, in the eighth version new categories of tumor size (≤ 2, 2–4, and > 4 vs. ≤ 2 and > 2 cm) and positive node number (0, 1–3, and ≥ 4 vs. 0 and ≥ 1) are incorporated into T and N staging, respectively [26, 33, 34]. However, after integrating these factors either as continuous or corresponding categorical variables into our nomogram, the performance did not markedly change. After transforming the SEER-18 staging data according to the eighth edition following Kamarajah et al. [26], the performance also remained very similar. Moreover, it will take considerable follow-up time for the survival associated with the new staging system to be adequately assessed. Therefore, our nomogram will still be applicable without compromised accuracy in the coming years.

Strengths of our study include the international population-based design, the largest number of patients with resected PaC ever investigated, the extensive potential prognostic factors studied, the *uniformly and consistently defined variables* especially TNM stage across countries, and the consistency and quality control in reporting through applying rigorous registry data standards. *Analyses were performed separately in each respective country without pooling*, which avoids the impact of the potential heterogeneity across countries.

Our work may have important clinical impacts and provides to our knowledge the first population-based model which can predict survival for patients with stage I–II PaC who underwent resection and chemotherapy. The model is robust, accurate, well-generalizable, practical, and easy-to-use. Our model may offer personalized patient survival estimates and facilitate clinical counseling for both patients and doctors. Having an idea about the estimated survival of a specific patient could influence plans on follow-up and surveillance (e.g., frequency and examination modality) and thus possibly guide resource allocation. For some proportion of the resected patients considering further treatment, the predicted survival might encourage receipt of further chemotherapy. The international validation assures that our model could be used for survival stratification in international studies.

Patients with resected PaC do not respond equally to chemotherapy, and accordingly, the calibration plots also suggest that individual survival varied greatly despite the relatively consistent comprehensive survival across countries. Our study will help to initially stratify this patient population into subgroups with discrepant survival, and might serve as a platform for developing further endeavors to understand factors associated with chemotherapy responses and survival in resected PaC, including precise, individualized, and personalized genomic and proteomic survivorship investigations.

Like any observational registry-based investigation, our study also has some limitations. Our model predicts survival at the average population level, and when applying this model in specific centers or regions with different care patterns, there could be some inconsistencies between predicted and actual survival. Nevertheless, as revealed by the calibration plots, the real-world survival was still in good accordance with the prediction for a single individual. Residual confounding is a concern. Some significant variables (e.g., tumor size) were only registered in certain databases. Differences in survival pattern across countries might be partly associated with variation in the prescription of chemotherapy and/or the underlying ethnic/racial distribution, even though association results remained similar after limiting the US cohort to white. Notably, there were some differences in patient and tumor characteristics across registries. For instance, in Slovenia, tumors were generally more advanced and poorly differentiated, and the actual survival was the lowest. Nevertheless, these variables were adjusted for in our multivariable analyses.

Population-based registries collected limited information on variables including family and patient health history and individual-level socioeconomic status. In addition, we were unable to determine the molecular or genetic subtype of PaC [16], which probably plays a role in prognosis and explains the moderate C-index of our nomogram. Accordingly, our nomogram is limited by failure to incorporate these and other recognized prognostic parameters (e.g., neurovascular invasion and type of chemotherapy). Further efforts on collection and incorporation of more relevant variables are encouraged to improve this model.

Notably, all known models predicting PaC survival perform very modestly [23, 24, 30–32]. Our nomogram with selection of only chemotherapy-treated resected PaC patients does not perform better compared to previous models with selection of all patients undergoing resection [23, 24, 30–32], which might limit the added value of the selection for the current nomogram. The lack of detailed information on chemotherapy which has not been routinely collected in most registries is another limitation of this population-based registry-based study. Collection of such information is strongly encouraged in future registration practice. During the study period, the type of chemotherapy was mainly gemcitabine monotherapy, followed by 5-fluorouracil-based therapy. The ESPAC-3 [46] and RTOG 97-04 randomized trials [47] demonstrated similar efficacy and effectiveness regarding survival between gemcitabine and 5-fluorouracil in the adjuvant setting. The landscape of systemic treatment (e.g., agent and formula) and treatment sequence for PaC are rapidly changing, which might limit the possible use of this nomogram.

Despite the moderate C-index, the agreement between predicted and actual survival was very good. All variables included in our nomogram are easily available in clinics, compared to the not routinely measured and costly molecular markers. It is herein the first time that the contributions of these risk factors are quantified and integrated into a single model for survival prediction in resected and chemotherapy-treated PaC with international validations.

## Conclusions

This large international population-based investigation revealed independent factors associated and not associated with survival in patients with resected stage I–II PaC receiving chemotherapy, with country-specific association patterns and strengths. We further established and internationally validated a novel, robust, and reliable survival-predicting model, which may provide the basis for more precise individualized survival estimation and which could be useful for clinical counseling. Our nomogram in line with all known models predicting survival in resected PaC performs modestly.

## Additional file

Additional file 1: **Table S1.** Selection of contacted national population-based cancer registries in Europe. **Table S2. **General information on participating population-based registries. **Table S3.** Inclusion and exclusion codes according to International Classification of Diseases for Oncology, Third Edition. **Table S4.** Comparison of the Memorial Sloan-Kettering Cancer Center nomogram with the nomogram established in this study for survival in resected pancreatic cancer for Western patient. (DOCX 33 kb)

## Abbreviations

HR: Hazard ratio
PaC: Pancreatic cancer
SEER: Surveillance, Epidemiology, and End Results

## References

[1] Torre LA, Bray F, Siegel RL, Ferlay J, Lortet-Tieulent J, Jemal A (2015). Global cancer statistics, 2012. CA Cancer J Clin.

[2] Khorana AA, Mangu PB, Berlin J, Engebretson A, Hong TS, Maitra A, Mohile SG, Mumber M, Schulick R, Shapiro M, et al. Potentially curable pancreatic Cancer: American Society of Clinical Oncology Clinical Practice Guideline Update. J Clin Oncol. 2017;35(20):2324–8. 10.1200/JCO.2017.72.4948 28398845

[3] Huang L, Jansen L, Balavarca Y, Molina-Montes E, Babaei M, van der Geest L, Lemmens V, Van Eycken L, De Schutter H, Johannesen TB (2019). Resection of pancreatic cancer in Europe and USA: an international large-scale study highlighting large variations. Gut.

[4] Huang L, Jansen L, Balavarca Y, Babaei M, van der Geest L, Lemmens V, Van Eycken L, De Schutter H, Johannesen TB, Primic-Zakelj M (2018). Stratified survival of resected and overall pancreatic cancer patients in Europe and the USA in the early twenty-first century: a large, international population-based study. BMC Med.

[5] Tempero MA, Malafa MP, Al-Hawary M, Asbun H, Bain A, Behrman SW, Benson AB, Binder E, Cardin DB, Cha C (2017). Pancreatic adenocarcinoma, version 2.2017, NCCN Clinical Practice Guidelines in Oncology. J Natl Compr Cancer Netw.

[6] Khorana AA, Mangu PB, Berlin J, Engebretson A, Hong TS, Maitra A, Mohile SG, Mumber M, Schulick R, Shapiro M (2016). Potentially curable pancreatic cancer: American Society of Clinical Oncology Clinical Practice Guideline. J Clin Oncol.

[7] Ducreux M, Cuhna AS, Caramella C, Hollebecque A, Burtin P, Goere D, Seufferlein T, Haustermans K, Van Laethem JL, Conroy T (2015). Cancer of the pancreas: ESMO Clinical Practice Guidelines for diagnosis, treatment and follow-up. Ann Oncol.

[8] Huang L, Jansen L, Balavarca Y, van der Geest L, Lemmens V, Van Eycken L, De Schutter H, Johannesen TB, Primic-Zakelj M, Zadnik V (2018). Non-surgical therapies for resected and unresected pancreatic cancer in Europe and USA in 2003-2014: a large international population-based study. Int J Cancer.

[9] Garcea G, Dennison AR, Pattenden CJ, Neal CP, Sutton CD, Berry DP (2008). Survival following curative resection for pancreatic ductal adenocarcinoma. A systematic review of the literature. JOP.

[10] Li D, Hu B, Zhou Y, Wan T, Si X (2018). Impact of tumor size on survival of patients with resected pancreatic ductal adenocarcinoma: a systematic review and meta-analysis. BMC Cancer.

[11] Schorn S, Demir IE, Haller B, Scheufele F, Reyes CM, Tieftrunk E, Sargut M, Goess R, Friess H, Ceyhan GO (2017). The influence of neural invasion on survival and tumor recurrence in pancreatic ductal adenocarcinoma - a systematic review and meta-analysis. Surg Oncol.

[12] Pindak D, Tomas M, Dolnik J, Duchon R, Pavlendova J (2017). Morbidity, mortality and long term survival in patients with vascular resection in pancreatic cancer - single center experience. Neoplasma.

[13] Yamamoto T, Yagi S, Kinoshita H, Sakamoto Y, Okada K, Uryuhara K, Morimoto T, Kaihara S, Hosotani R (2015). Long-term survival after resection of pancreatic cancer: a single-center retrospective analysis. World J Gastroenterol.

[14] Jouffret L, Turrini O, Ewald J, Moutardier V, Iovanna JL, Delpero JR (2015). Long-term survivors after pancreatectomy for cancer: the TNM classification is outdated. ANZ J Surg.

[15] Erdmann JI, Morak MJ, Duivenvoorden HJ, van Dekken H, Kazemier G, Kok NF, van Eijck CH (2015). Long-term survival after resection for non-pancreatic periampullary cancer followed by adjuvant intra-arterial chemotherapy and concomitant radiotherapy. HPB (Oxford).

[16] Cancer Genome Atlas Research Network, Electronic address aadhe, Cancer genome atlas research N (2017). Integrated genomic characterization of pancreatic ductal adenocarcinoma. Cancer Cell.

[17] Yurgelun MB, Chittenden AB, Morales-Oyarvide V, Rubinson DA, Dunne RF, Kozak MM, Qian ZR, Welch MW, Brais LK, Da Silva A (2019). Germline cancer susceptibility gene variants, somatic second hits, and survival outcomes in patients with resected pancreatic cancer. Genet Med.

[18] Lawrence YR, Moughan J, Magliocco AM, Klimowicz AC, Regine WF, Mowat RB, DiPetrillo TA, Small W, Simko JP, Golan T (2018). Expression of the DNA repair gene MLH1 correlates with survival in patients who have resected pancreatic cancer and have received adjuvant chemoradiation: NRG Oncology RTOG Study 9704. Cancer.

[19] Mahajan UM, Langhoff E, Goni E, Costello E, Greenhalf W, Halloran C, Ormanns S, Kruger S, Boeck S, Ribback S (2018). Immune cell and stromal signature associated with progression-free survival of patients with resected pancreatic ductal adenocarcinoma. Gastroenterology.

[20] Yan H, Qiu W, Koehne de Gonzalez AK, Wei JS, Tu M, Xi CH, Yang YR, Peng YP, Tsai WY, Remotti HE (2019). HHLA2 is a novel immune checkpoint protein in pancreatic ductal adenocarcinoma and predicts post-surgical survival. Cancer Lett.

[21] Sideras K, Biermann K, Yap K, Mancham S, Boor PPC, Hansen BE, Stoop HJA, Peppelenbosch MP, van Eijck CH, Sleijfer S (2017). Tumor cell expression of immune inhibitory molecules and tumor-infiltrating lymphocyte count predict cancer-specific survival in pancreatic and ampullary cancer. Int J Cancer.

[22] Balachandran VP, Gonen M, Smith JJ, DeMatteo RP (2015). Nomograms in oncology: more than meets the eye. Lancet Oncol.

[23] Brennan MF, Kattan MW, Klimstra D, Conlon K (2004). Prognostic nomogram for patients undergoing resection for adenocarcinoma of the pancreas. Ann Surg.

[24] Tol JA, Brosens LA, van Dieren S, van Gulik TM, Busch OR, Besselink MG, Gouma DJ (2015). Impact of lymph node ratio on survival in patients with pancreatic and periampullary cancer. Br J Surg.

[25] Surveillance, Epidemiology, and End Results (SEER) Program (www.seer.cancer.gov) Research Data (1973–2015), National Cancer Institute, DCCPS, Surveillance Research Program, released April 2018, based on the November 2017 submission.

[26] Kamarajah SK, Burns WR, Frankel TL, Cho CS, Nathan H (2017). Validation of the American Joint Commission on Cancer (AJCC) 8th Edition Staging System for Patients with Pancreatic Adenocarcinoma: a Surveillance, Epidemiology and End Results (SEER) analysis. Ann Surg Oncol.

[27] Hess KR (1995). Graphical methods for assessing violations of the proportional hazards assumption in Cox regression. Stat Med.

[28] Harrell FE, Lee KL, Mark DB (1996). Multivariable prognostic models: issues in developing models, evaluating assumptions and adequacy, and measuring and reducing errors. Stat Med.

[29] Hanley JA, McNeil BJ (1983). A method of comparing the areas under receiver operating characteristic curves derived from the same cases. Radiology.

[30] de Castro SM, Biere SS, Lagarde SM, Busch OR, van Gulik TM, Gouma DJ (2009). Validation of a nomogram for predicting survival after resection for adenocarcinoma of the pancreas. Br J Surg.

[31] Clark EJ, Taylor MA, Connor S, O'Neill R, Brennan MF, Garden OJ, Parks RW (2008). Validation of a prognostic nomogram in patients undergoing resection for pancreatic ductal adenocarcinoma in a UK tertiary referral centre. HPB (Oxford).

[32] Ferrone CR, Kattan MW, Tomlinson JS, Thayer SP, Brennan MF, Warshaw AL (2005). Validation of a postresection pancreatic adenocarcinoma nomogram for disease-specific survival. J Clin Oncol.

[33] Schlitter AM, Jesinghaus M, Jager C, Konukiewitz B, Muckenhuber A, Demir IE, Bahra M, Denkert C, Friess H, Kloeppel G (2017). pT but not pN stage of the 8th TNM classification significantly improves prognostication in pancreatic ductal adenocarcinoma. Eur J Cancer.

[34] Allen PJ, Kuk D, Castillo CF, Basturk O, Wolfgang CL, Cameron JL, Lillemoe KD, Ferrone CR, Morales-Oyarvide V, He J (2017). Multi-institutional validation study of the American Joint Commission on Cancer (8th edition) changes for T and N staging in patients with pancreatic adenocarcinoma. Ann Surg.

[35] Schnelldorfer T, Ware AL, Sarr MG, Smyrk TC, Zhang L, Qin R, Gullerud RE, Donohue JH, Nagorney DM, Farnell MB (2008). Long-term survival after pancreatoduodenectomy for pancreatic adenocarcinoma: is cure possible?. Ann Surg.

[36] Kuhlmann KF, de Castro SM, Wesseling JG, ten Kate FJ, Offerhaus GJ, Busch OR, van Gulik TM, Obertop H, Gouma DJ (2004). Surgical treatment of pancreatic adenocarcinoma; actual survival and prognostic factors in 343 patients. Eur J Cancer.

[37] Huebner M, Kendrick M, Reid-Lombardo KM, Que F, Therneau T, Qin R, Donohue J, Nagorney D, Farnell M, Sarr M (2012). Number of lymph nodes evaluated: prognostic value in pancreatic adenocarcinoma. J Gastrointest Surg.

[38] Murakami Y, Uemura K, Sudo T, Hayashidani Y, Hashimoto Y, Nakashima A, Yuasa Y, Kondo N, Ohge H, Sueda T (2010). Number of metastatic lymph nodes, but not lymph node ratio, is an independent prognostic factor after resection of pancreatic carcinoma. J Am Coll Surg.

[39] Hammel P, Huguet F, van Laethem JL, Goldstein D, Glimelius B, Artru P, Borbath I, Bouche O, Shannon J, Andre T (2016). Effect of chemoradiotherapy vs chemotherapy on survival in patients with locally advanced pancreatic cancer controlled after 4 months of gemcitabine with or without erlotinib: the LAP07 Randomized Clinical Trial. JAMA.

[40] Luberice K, Downs D, Sadowitz B, Ross S, Rosemurgy A (2017). Has survival improved following resection for pancreatic adenocarcinoma?. Am J Surg.

[41] Benassai G, Quarto G, Perrotta S, Furino E, Benassai GL, Amato B, Bianco T, De Palma G, Forestieri P (2015). Long-term survival after curative resection for pancreatic ductal adenocarcinoma--surgical treatment. Int J Surg.

[42] Konstantinidis IT, Warshaw AL, Allen JN, Blaszkowsky LS, Castillo CF, Deshpande V, Hong TS, Kwak EL, Lauwers GY, Ryan DP (2013). Pancreatic ductal adenocarcinoma: is there a survival difference for R1 resections versus locally advanced unresectable tumors? What is a "true" R0 resection. Ann Surg.

[43] Tempero MA, Malafa MP, Behrman SW, Benson AB, Casper ES, Chiorean EG, Chung V, Cohen SJ, Czito B, Engebretson A (2014). Pancreatic adenocarcinoma, version 2.2014: featured updates to the NCCN guidelines. J Natl Compr Cancer Netw.

[44] Chandrasegaram MD, Goldstein D, Simes J, Gebski V, Kench JG, Gill AJ, Samra JS, Merrett ND, Richardson AJ, Barbour AP (2015). Meta-analysis of radical resection rates and margin assessment in pancreatic cancer. Br J Surg.

[45] Butturini G, Stocken DD, Wente MN, Jeekel H, Klinkenbijl JH, Bakkevold KE, Takada T, Amano H, Dervenis C, Bassi C (2008). Influence of resection margins and treatment on survival in patients with pancreatic cancer: meta-analysis of randomized controlled trials. Arch Surg.

[46] Neoptolemos JP, Stocken DD, Bassi C, Ghaneh P, Cunningham D, Goldstein D, Padbury R, Moore MJ, Gallinger S, Mariette C (2010). Adjuvant chemotherapy with fluorouracil plus folinic acid vs gemcitabine following pancreatic cancer resection: a randomized controlled trial. JAMA.

[47] Regine WF, Winter KA, Abrams R, Safran H, Hoffman JP, Konski A, Benson AB, Macdonald JS, Rich TA, Willett CG (2011). Fluorouracil-based chemoradiation with either gemcitabine or fluorouracil chemotherapy after resection of pancreatic adenocarcinoma: 5-year analysis of the U.S. Intergroup/RTOG 9704 phase III trial. Ann Surg Oncol.

