# Supplementary material for: Development and validation of a prognostic model to predict the prognosis of patients who underwent chemotherapy and resection of pancreatic adenocarcinoma: a large international population-based cohort study
Source: BMC Med. 2019 Mar 25;17:66. doi: 10.1186/s12916-019-1304-y (PMC6432746; doi:10.1186/s12916-019-1304-y)
Supplement: Supplementary file 1 — Table S1. Selection of contacted national population-based cancer registries in Europe. Table S2. General information on participating population-based registries. Table S3. Inclusion and exclusion codes according to International Classification of Diseases for Oncology, Third Edition. Table S4. Comparison of the Memorial Sloan-Kettering Cancer Center nomogram with the nomogram established in this study for survival in resected pancreatic cancer for Western patient. (DOCX 33 kb) [file 12916_2019_1304_MOESM1_ESM.docx]

**Supplementary materials**

**Item no.:** 5

**Supplementary Results**

**Table S1.** Selection of contacted national population-based cancer registries in Europe

**Table S2.** General information on participating population-based registries

**Table S3.** Inclusion and exclusion codes according to International Classification of Diseases for Oncology, Third Edition

**Table S4.** Comparison of the Memorial Sloan-Kettering Cancer Center nomogram with the nomogram established in this study for survival in resected pancreatic cancer for Western patients

**Supplementary Results**

***Model function***

Reference values: sex="Female", t="T1", n="N0", dgrade="Well" (the variable names “age”, “sex”, “t”, “n”, and “dgrade” are for “Age”, “Sex”, “T Stage”, “N Stage”, and “Differentiation” in the nomogram (**Figure 2**), respectively)

Function: -0.71148656+0.09222951*(sex=="Male")-0.0080361358*age+4.0504221e-05*pmax(age-47,0)^3-0.00019415844*pmax(age-59,0)^3+0.00026940813*pmax(age-65,0)^3-0.00014449267*pmax(age-71,0)^3+2.8738757e-05*pmax(age-80,0)^3+0.27621944*(t=="T2")+0.410323*(t=="T3")+0.42855775*(n=="N1")+0.24623505*(dgrade=="Intermediate")+0.50857222*(dgrade=="Poor/undifferentiated")

**Table S1.** Selection of contacted national population-based cancer registries in Europe^1^

| Country of contacted registry | Included | Comment if not included |
| --- | --- | --- |
| *Norther Europe* |  |  |
| Finland | No | Surgical treatment not validated |
| Sweden | No | National data not statistically validated |
| Norway | Yes |  |
| Iceland | No | No national population-based data on treatment |
| Denmark | No | Participation consent withdrawn due to legislation issues |
| *Western Europe* |  |  |
| The UK | No | No ready-to-use national population-based data on treatment or TNM stage |
| Ireland | No | No further response after initial contact |
| The Netherlands | Yes |  |
| Belgium | Yes |  |
| *Southern Europe* |  |  |
| Bulgaria | No | No national population-based data on treatment |
| Serbia | No | No response |
| Slovenia | Yes |  |
| Croatia | No | No national population-based data on surgical treatment |
| *Eastern Europe* |  |  |
| Estonia | No | Small number of resected cases and short incidence periods recorded not allowing for robust survival analysis |
| Latvia | No | No national population-based data on treatment |
| Lithuania | No | No response |
| Ukraine | No | Insufficient resources for data collection |
| Slovakia | No | No response |
| *Central Europe* |  |  |
| Poland | No | No response |
| Czech Republic | No | No national population-based data on treatment |
| Austria | No | No national population-based data on treatment |

^1^For the other countries and regions in Europe (*e.g.*, France, Italy, and Germany) not listed in this table, no corresponding national population-based registries were found through careful search.

**Table S2.** General information on participating population-based registries

| Source | Country | Diagnosis period | Censoring date | Registered cases^2^ |  | Excluded cases^1^ | | | |  | Analyzed cases | Follow-up months^3^ |
| --- | --- | --- | --- | --- | --- | --- | --- | --- | --- | --- | --- | --- |
|  |  |  |  |  | DCO/  autopsy | Not resected | No/unknown chemotherapy | Not microscopically-confirmed/ineligible pathology | Stage 0/III/  IV/unknown | Survival <3 months/unknown |  |  |
| SEER-18^4^ | The US | Jan. 2004-Dec. 2015 | Dec. 31, 2015 | 122826 | 3917 | 99448 | 7076 | 1102 | 1436 | 328 | 9519 | 56 (28-89) |
| BCR | Belgium | Jan. 2004-Dec. 2013 | Jul. 1, 2015 | 12146 | NA | 9501 | 1157 | 142 | 233 | 8 | 1105 | 64 (40-89) |
| NCR | The Netherlands | Jan. 2003-Dec. 2014 | Feb. 1, 2015 | 22579 | 99 | 19534 | 1743 | 106 | 86 | 29 | 982 | 36 (20-59) |
| CRS | Slovenia | Jan. 2003-Dec. 2013 | May 25, 2016 | 3376 | 54 | 2651 | 489 | 22 | 42 | 0 | 118 | 75 (51-87) |
| CRN | Norway | Jan. 2003-Dec. 2014 | Jun. 30, 2015 | 8022 | 333 | 6471 | 1000 | 46 | 59 | 0 | 113 | 40 (32-65) |

^1^Data exclusion followed this sequence: DCO/autopsy, not resected, no/unknown chemotherapy, stage 0/III/IV, not microscopically-confirmed/ineligible pathology, and survival <3 months/unknown (from left to right).

^2^A preliminary data-cleaning process had been performed to exclude cases with ineligible histology types except cystic, mucinous, and serous malignancies.

^3^Shown as median (interquartile range), and computed using the reverse Kaplan-Meier method.

^4^Data of the year 2003 was not analyzed, as the TNM stage (version 6/7) information was unavailable.

SEER, [Surveillance, Epidemiology, and End Results Program](http://seer.cancer.gov/); BCR, Belgian Cancer Registry; NCR, Netherlands Cancer Registry; CRS, Cancer Registry of Slovenia; CRN, Cancer Registry of Norway; DCO, death certificate only; NA, not available.

**Table S3.** Inclusion and exclusion codes according to International Classification of Diseases for Oncology, Third Edition^1^

| **Category** |  | **Code** |
| --- | --- | --- |
| **Topology** | Inclusion | C25.0, C25.1, C25.2, C25.3, C25.7, C25.8, C25.9 |
|  | Exclusion | C25.4 |
| **Morphology**^1^ | Inclusion | 8140-8389 (adenomas and adenocarcinomas), 8500-8549 (ductal and lobular neoplasms) |
|  | Exclusion | 8000-8009 (unspecified neoplasms), 8010-8049 (epithelial neoplasms, NOS), 8050-8089 (squamous cell neoplasms), 8150-8153, 8155-8157, 8160, 8162, 8170, 8180, 8240-8243, 8246-8249, 8312, 8440-8499 (cystic and mucinous neoplasms), 8550-8559 (acinar cell neoplasms), 8560-8579 (complex epithelial neoplasms), 8680, 8700, 8800-8802, 8810, 8825, 8830, 8851, 8852, 8858, 8890, 8891, 8900, 8920, 8936, 8982, 9043, 9100, 9120, 9250, 9364, 9473, 9500, 9591, 9673, 9680, 9687, 9691, 9695, 9702 |
| **Behavior** | Inclusion | 3 |
|  | Exclusion | 0, 2 |

^1^http://codes.iarc.fr/

^2^Partly based on [Surveillance, Epidemiology, and End Results Program](http://seer.cancer.gov/) broad groupings.

**Table S4.** Comparison of the Memorial Sloan-Kettering Cancer Center nomogram with the nomogram established in this study for survival for Western patients with resected pancreatic cancer

| Nomogram | Dataset | Authors | Cohort origin | Publication year | Study design | Resection period | Follow-up end | Sample size | Survival index | Additional factors | Backward selection | Concordance index | 95% confidence interval | External validation |
| --- | --- | --- | --- | --- | --- | --- | --- | --- | --- | --- | --- | --- | --- | --- |
| The one established in this study | Training | Huang *et al.* | The US | - | Population-based | 2004-2015 | 2015 | 9519 | Median survival time, 1-/2-/3-/5-year survival rate | - | Yes | 0.60 | 0.59-0.61 | - |
|  | Validation | Huang *et al.* | Belgium, The Netherlands, Norway, Slovenia | - | Multinational population-based | 2003-2014 | 2016 | 2318 | Median survival time, 1-/2-/3-/5-year survival rate | - | - | 0.58-0.63 | Shown in **Table 4** | Accurate |
| Memorial Sloan-Kettering Cancer Center | Training | Brennan *et al.* | The US | 2004 | Single institutional | 1983-2000 | 2002 | 555 | 3-year survival | Portal vein resection, splenectomy, resection margin, back pain, weight loss, maximum pathologic axis | Unspecified | 0.64 | Not reported | - |
|  | Validation I | Ferrone *et al.* | The US | 2005 | Single institutional | 1985-2003 | Not reported | 375 | 3-year survival | - | - | 0.62 | Not reported | Accurate |
|  | Validation II | Clark *et al.* | UK | 2008 | Single institutional | 1995-2005 | Not reported | 63 | 3-year survival | - | - | Not reported | Not reported | Not accurate |
|  | Validation III | de Castro *et al.* | The Netherlands | 2009 | Single institutional | 1985-2004 | 2007 | 263 | 3-year survival | - | - | 0.61 | Not reported | Accurate |

-, not available.
